# Selenium Concentration and Speciation in Formulae for Infants: Implications for Infant Health

**DOI:** 10.3390/foods15173007

**Published:** 2026-08-26

**Authors:** Małgorzata Dobrzyńska, Zofia Wojciechowska, Anna Szczepańska-Álvarez, Katarzyna A. Kaczmarek-Kryszak, Juliusz Przysławski, Przemysław Niedzielski, Sławomira Drzymała-Czyż

**Affiliations:** 1 Department of Bromatology, Poznan University of Medical Sciences, Rokietnicka 3 Street, 60-806 Poznan, Poland; kaczmarek.kryszak@gmail.com (K.A.K.-K.); jprzysla@ump.edu.pl (J.P.); drzymala@ump.edu.pl (S.D.-C.); 2 Department of Analytical Chemistry, Faculty of Chemistry, Adam Mickiewicz University, Uniwersytetu Poznanskiego 8 Street, 61-614 Poznan, Poland; zofia.wojciechowska@amu.edu.pl (Z.W.); przemyslaw.niedzielski@amu.edu.pl (P.N.); 3 Department of Mathematical and Statistical Methods, Poznan University of Life Sciences, 60-637 Poznan, Poland; anna.szczepanska-alvarez@up.poznan.pl; 4 Doctoral School, Poznan University of Medical Sciences, Bukowska 70 Street, 60-812 Poznan, Poland

**Keywords:** infant nutrition, infant formula, follow-up formula, Se forms, toxic, selenomethionine, methylselenocysteine

## Abstract

Inappropriate concentrations of elements in formulae for infants may lead to adverse health effects. Selenium (Se) is a trace element essential for the proper development of infants. It is naturally present in human milk and is also added to infant formulae. However, both insufficient and excessive Se intake may be harmful. The aim of this study was to determine Se concentrations and chemical forms in 148 formulae for infants available on the Polish market and to assess their nutritional safety. Se concentrations were determined using inductively coupled plasma mass spectrometry (ICP-iCRC-MS). Se speciation was performed using a HPLC system coupled to ICP-iCRC-MS. The Se concentrations in most analyzed formulae were in good agreement with the recommended EU limits (median 4.5 µg/100 kcal, range 0–15.6 µg/100 kcal). However, the estimated daily intake for 6-month-old infants exceeded the tolerable upper intake level in six formulae, indicating a potential health risk. Speciation analysis showed that the dominant Se forms were sodium selenite and sodium selenate, and their levels were mostly consistent with the manufacturers’ declarations. Additionally, trace amounts of selenometionine (SeMet) and methyloselenocysteine (MeSeCys) were detected. Therefore, regular monitoring of the composition of infant formulae remains necessary. The detection of SeMet and MeSeCys provides new avenues for research on Se content and Se speciation in formulae intended for infants.

## 1. Introduction

Infant nutrition during the first year of life plays a fundamental role in ensuring proper growth and development. According to leading international authorities, such as the European Society for Pediatric Gastroenterology, Hepatology and Nutrition (ESPGHAN), European Food Safety Authority (EFSA), the American Society for Par-enteral and Enteral Nutrition (ASPEN), and the Food and Drug Administration (FDA), breastfeeding is considered the gold standard for infant nutrition in the first months of life [1,2]. However, there are specific medical contraindications to breastfeeding. In infants, classic galactosemia is the only condition that completely precludes breastfeeding, while phenylketonuria allows partial breastfeeding in combination with an appropriate low-phenylalanine formula, under close clinical supervision. Breastfeeding should be avoided in cases of HTLV-1 or HTLV-2 infection, a serious illness that prevents safe infant care, or when the mother requires medications that are inappropriate for breastfeeding, including radioisotopes or certain chemotherapeutic or immunosuppressive drugs [3]. Despite these clinical considerations, formula feeding continues to constitute a substantial proportion of infant nutrition worldwide, although only a minority of infants require it due to medical conditions. According to World Health Organization (WHO) report from 2025, only 47% of infants under 6 months of age are exclusively breastfed [4]. Therefore, public health initiatives promoting breastfeeding should continue to be strengthened. At the same time, when formula feeding is necessary, efforts should be made to ensure that infant formulae resemble human milk as closely as possible [5].

The composition of infant formula is strictly regulated. In the European Union, nutrient requirements and permitted chemical forms are defined by EU legislation [6,7]. In the United States, the composition of infant formula is regulated exclusively by the FDA [8]. Recent developments in the field include the introduction of probiotics and human milk oligosaccharides (HMOs) into formulae, as well as the increasing availability of specialized products, such as formulae for premature infants or those with lactose intolerance [9]. Although this broadens the range of available options, it also poses challenges for quality control. In Poland, approximately 150 formulae intended for infants up to 12 months of age are currently available [10]. These include infant formulae intended for infants aged 0–6 months, follow-on formulae for infants aged 6–12 months, and formulae for special medical purposes. The use of formulae for special nutritional purposes is strictly defined by the manufacturer on the label.

From a clinical perspective, monitoring the content of nutrients in formula for infants and their potential impact on infant health and development is essential. Trace elements are of particular interest, as they are required in very small amounts yet play key physiological roles [11]. Importantly, not only the total amount of elements but also their chemical form and oxidation state influence their biological function. One of the most illustrative examples is selenium (Se) [12].

According to European Union and FDA regulations, only two inorganic forms of Se are permitted in infant formulae: sodium selenite and sodium selenate. Regardless of formula type, the Se content should range from 3.0 to 8.6 µg/100 kcal [6,7].

It is worth emphasizing that both insufficient and excessive Se intake may be harmful. Se is essential for the proper functioning of the immune [13], endocrine [14], and reproductive systems [15]. Its immunoregulatory functions include modulation of cytokine production and maintenance of redox homeostasis by limiting oxidative stress [16,17]. This element is also required for thyroid hormone metabolism, particularly for the conversion of thyroxine (T4) to triiodothyronine (T3) [18]. Se deficiency in infants most commonly manifests as impaired growth, increased susceptibility to infections due to reduced glutathione peroxidase activity, and disturbances in thyroid hormone metabolism [19,20]. More specific clinical signs include erythrocyte macrocytosis and dermatologic changes (e.g., fingernail-bed abnormalities, pseudoalbinism), which occur less frequently but are characteristic of more pronounced deficiency [21]. Severe outcomes such as cardiomyopathy, muscle weakness, and heart failure are rare [22]. Excessive Se intake may lead to diarrhea, vomiting, and irritability [23]. Chronic excessive intake leads to selenosis, typically associated with organic Se forms, which manifests as hair and nail abnormalities, halitosis, muscle pain, memory impairment, mood disturbances, and severe paresthesia [24,25,26]. According to the available literature, both Se deficiency and toxicity in infants are rare, although potentially dangerous [19].

Therefore, the aim of this study was to determine the concentration and chemical forms of Se in infant formulae and assess their safety. In total, 148 samples were analyzed in this study.

## 2. Materials and Methods

### 2.1. Materials

**Figure 1:**
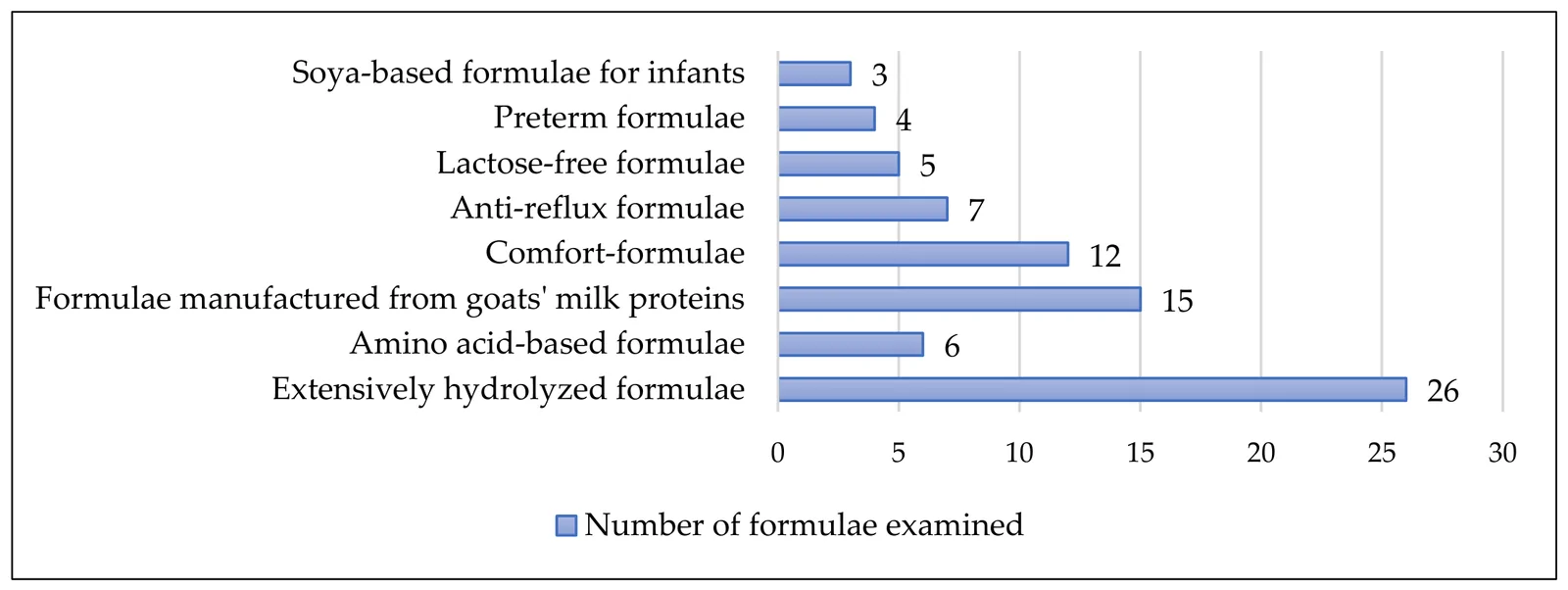
Detailed types of special medical purpose formulae included in the analysis.

A total of 148 powdered milk formulae available on the Polish market between 2019 and 2023, intended for consumption during the first year of life, were subjected to analysis. The sample encompassed 37 infant formulae, 33 follow-on formulae, and 78 formulae for special medical purposes. The detailed types of special medical purpose formulae analysed are presented in Figure 1. A total of 22 different brands were included, and products that changed their declared composition during the study period were treated as separate formulae. All samples were collected, coded, and stored according to the manufacturers’ instructions, and analyzed together in a single analytical batch to minimize inter-batch variability.

Validation of the analytical procedures was performed using certified reference material—skimmed milk powder ERM-BD150 (European Reference Materials, Geel, Belgium). Depending on the type of formula, one to three parallel samples (from independent commercial containers) were analysed: four formulae were tested in duplicate, twenty in triplicate, and the remaining in a single replicate. The number of repetitions was determined by both the availability of the products and the financial constraints of the study.

### 2.2. Methods

#### 2.2.1. Total Se Concentrations

For total Se determination, 0.25 g of milk samples were mineralized in 5 mL of 65% nitric acid (Merck, Darmstadt, Germany) in closed PTFE containers in the Mars 6 Microwave Digestion System (CEM, Matthews, USA). The program included 20 min of heating to 180 °C (ramp), 20 min of holding the temperature and 30 min of cooling. Afterward, samples were poured over to the polypropylene Falcon tubes and filled up to final volume (15 mL) with ultra-pure water with a resistivity ≥18 MΩ cm (Milli-Q system, Merck Millipore, Darmstadt, Germany). A commercial Se standard (Romil, Cambridge, UK) was used for calibration of total Se determination. High-purity argon and hydrogen gases (N–5.0, purity 99.999%) were supplied by Linde (Warsaw, Poland) and used throughout the ICP MS analysis.

Total Se concentrations were measured using inductively coupled plasma mass spectrometry with Integrated Collision Reaction Cell ICP(iCRC)MS Plasma Quant MS Q (Analytik Jena, Jena, Germany). Interfering polyatomic ions were reduced with the use of iCRC with hydrogen as reaction gas before they reach the mass analyzer. Signal was measured in 5 replicates of 20 scans. Detection limits (DLs) were calculated using 3–sigma criteria.

#### 2.2.2. Selenium Speciation Analysis

For the Se species determination, 0.50 g of milk sample and 0.020 g of protease (Streptomyces griseus type XIV, Sigma Aldrich, St. Louis, MO, USA) were weighed into a test tube, followed by the addition of 7 mL of water. The extraction was performed in an ultrasonic bath for 30 min at ambient temperature. Then, the samples were centrifuged for 10 min using the MPW-260R laboratory centrifuge (MPW Med. Instruments, Warszawa, Poland). Supernatants were filtered using a paper filter, washed with 200 mL of ultra-pure water, transferred to 10 mL test tubes, and diluted to a final volume of 10 mL with 5 mM/L acetate buffer.

Stock standard solutions (1000 mg Se/L in a single solution) of selenite (Se(IV)), selenate (Se(VI)), selenomethionine (SeMet) and methylselenocysteine (MeSeCys) were prepared by dissolving appropriate amounts of disodium selenite (Na_2_SeO_3_), disodium selenate (Na_2_SeO_4_), seleno L methionine, and (methyl)selenocysteine hydrochloride (Sigma Aldrich, USA). Stock solutions were stored in plastic bottles at 4 °C. Less concentrated standard solutions were prepared by diluting the stock solutions and were prepared daily.

The mobile phase for chromatographic separation was prepared by dissolving 5.4 g of CH_3_COONH_4_ (Sigma Aldrich, St. Louis, MO, USA) and mixing it with 4.6 mL of CH_3_COOH (Stanlab, Lublin, Poland), followed by dilution to 1 L with ultrapure water to obtain mobile phase B (150 mM acetate buffer). This stock solution was further diluted to 5 mM to obtain mobile phase A.

The speciation analysis was performed using a PQ LC system (Analytik Jena, Jena, Germany) coupled to the ICP(iCRC)MS. The HPLC system consisted of an S 1130 HPLC pump (Analytik Jena, Jena, Germany), an S 5300 autosampler (Analytik Jena, Jena, Germany), and an anion-exchange column PRP-X100 (50 mm × 4.1 mm i.d., 5 μm; Hamilton Company, Bonaduz, Switzerland). Standards and samples were injected using a 100 μL sample loop.

Chromatographic separation was performed with the column temperature set to 20 °C. Separation of Se compounds was achieved using a gradient flow with 5 mM and 150 mM acetate buffer. The flow rate of the mobile phase was maintained at 1.0 and 1.5 mL/min. The total separation time was about 9.5 min. Se species identification was based on retention time and signals at *m*/*z* 78, with additional monitoring at *m*/*z* 82. Quantification was based on external standard curves.

HPLC and ICP(iCRC)MS operating conditions are presented in the Supplemental Materials (Table S1).

#### 2.2.3. Analytical Figures of Merit

The analytical figures of merit for Se speciation by HPLC-ICP MS and total Se determination by ICP MS are summarized in Table 1. For quality control of the analysis, skimmed milk powder BD150 and wheat flour (BC210a, European Reference Materials, Belgium) were analyzed. The acceptable recovery (80–120%) with the certified total Se values was obtained for both CRMs. In addition to total Se, ERM BC210a has certified SeMet content. Recovery of the certified SeMet values was 47% using the extraction procedure applied in this study. The relatively low agreement obtained for SeMet in ERM-BC210a (47%), despite quantitative recovery of total Se, may be attributed to incomplete release of protein-bound SeMet in its free form under the applied extraction conditions. SeMet may have been transferred to the extract as a component of soluble proteins or peptides and thus included in the total Se measured directly by ICP MS without being quantified as free SeMet by HPLC–ICP MS. Independent analysis was performed with spike standard addition, obtaining satisfactory recoveries. The combined expanded uncertainty for total Se determination was less than 20% for the whole analytical procedure (a coverage factor k = 2 for approximately 95% confidence).

The median extraction efficiency for all analyzed samples, calculated as the ratio of total Se in the extract to total Se determined in HNO_3_ digestion, was 84% (1st—72%, 3rd—100%). The median speciation mass balance, calculated as the sum of the quantified Se species quantified by HPLC-ICP MS relative to total Se measured by ICP MS in the corresponding extract, was 77% (1st—51%, 3rd—92%). The “others” fraction was defined as the calculation residual and was calculated as total Se in extract minus sum of the quantified Se species.

**Figure 2:**
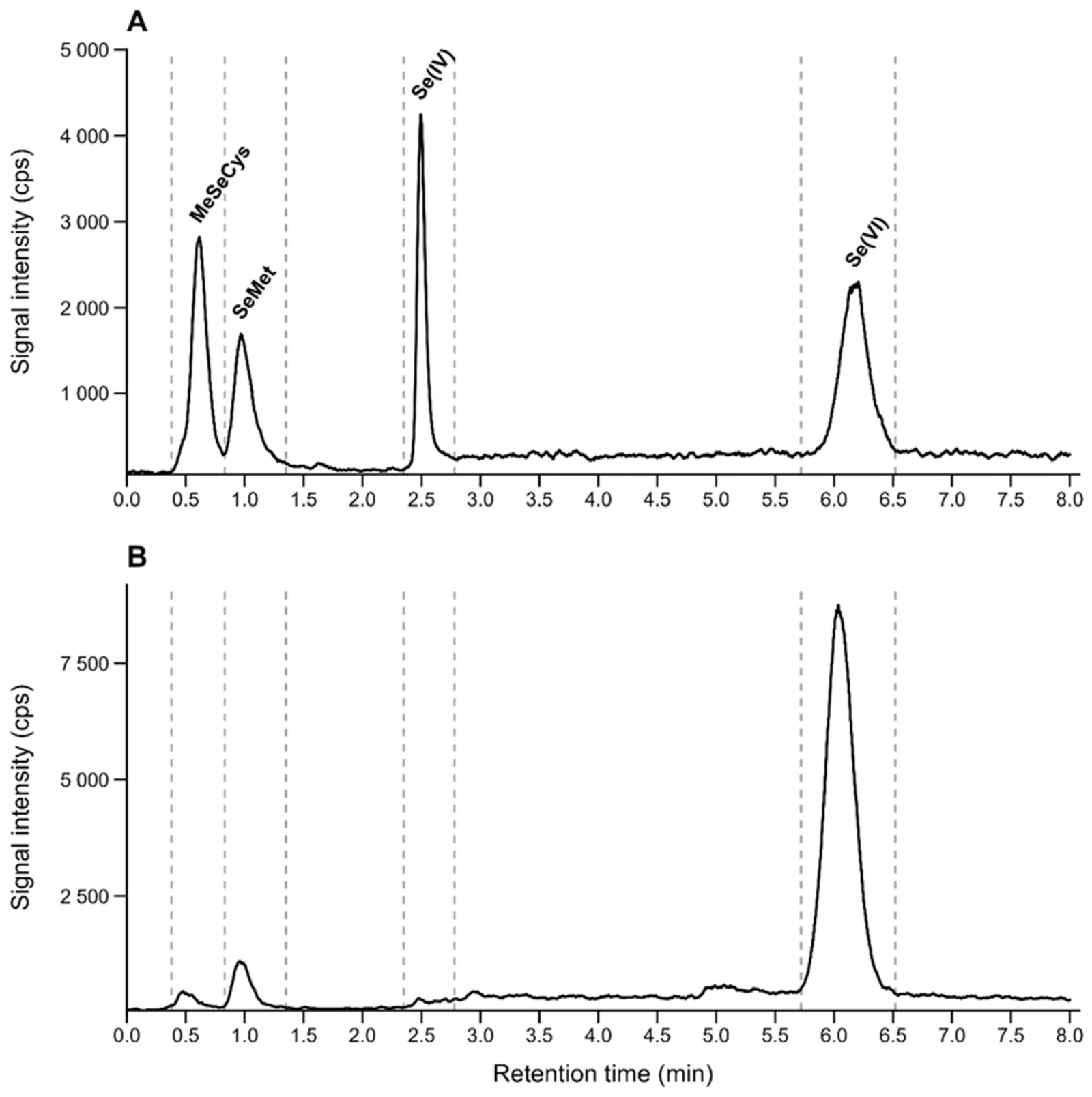
Chromatograms of (**A**) a standard containing 2.5 μg/L of each studied Se form and (**B**) an exemplary infant formulae sample.

Chromatographic resolution was evaluated using seven replicate injections of a mixed standard containing 10 µg/L of each Se species. Resolution was calculated from the retention times and peak widths at half height. The mean and standard deviation resolution values were 1.62 ± 0.09 for MeSeCys/SeMet, 9.01 ± 0.64 for SeMet/SeIV and 14.71 ± 0.82 for SeIV/SeVI. Representative chromatograms of Se species standard and an infant formulae sample are shown in Figure 2.

#### 2.2.4. Data Analyses

The Se concentrations were evaluated against the minimum and maximum levels permitted in formulae for infants according to EU regulations, namely Commission Directive 2016/127 and Commission Delegated Regulation (EU) 2016/128, both supplementing Regulation (EU) No. 609/2013 concerning compositional and information requirements for infant formula, follow-on formula, and food for special medical purposes [6,7]. The analytical values were converted to µg per 100 kcal and stratified into three formula types: infant formulae, follow-on formulae, and formulae for special medical purposes.

The estimated daily intake (EDI) of Se was calculated using the Se concentration in the formula, the infant’s daily energy requirement, and the amount of formula needed to meet that requirement, accounting for the energy density of the prepared formula. The EDI was calculated for all analyzed formulae. For products intended for infants from birth to 6 months (infant formulae), the EDI was determined for infants aged 1, 3, and 6 months. For formulae designed for infants aged 7–12 months (follow-on formulae), the EDI was calculated for infants aged 7 and 10 months. Formulae for special medical purposes were additionally subdivided into four categories according to the age range for which they were intended. In products formulated for infants from birth to 6 months, the EDI was calculated using the same age points as for standard infant formulae (1, 3, and 6 months). For formulae intended for infants aged 7–12 months, the EDI corresponded to the age points applied to follow-on formulae (7 and 10 months). For formulae intended for infants from birth to 12 months, the EDI was calculated for infants aged 1, 3, 6, 7, and 10 months. For formulae intend for preterm infants until achieving appropriate body weight, the EDI was calculated for infants aged 1, 3 and 6 months.

Recommendations on energy intake were applied to estimate the EDI of Se using the present data from the Stan et al. study, while average body weights were determined based on the recent WHO growth percentiles for infants [27]. It should be noted that, after 6 months of age, infants no longer meet their full energy requirements solely from formula due to the introduction of complementary foods. Therefore, it was assumed that infants at 7 months of age obtain 45% of their total energy intake from formula, and those at 10 months obtain 25% [28].

The results were compared with the nutritional guidelines applicable in Poland, as per the EFSA. The EDI was evaluated against the adequate intake (AI) for infants aged 6–11 months (20 µg/day) and the tolerable upper intake level (UL), set at 45 µg/day for infants aged 4–6 months and 55 µg/day for infants aged 7–11 months [29,30].

The identified Se species were expressed as percentages, taking into account the distribution of formulae according to their type: infant formulae, follow-on formulae, and formulae for special medical purposes. Due to differences in the ingredients used in the production of each formula category, the formulae for special medical purposes were further subdivided into the following groups: extensively hydrolyzed formulae, amino acid-based formulae, goat milk-based formulae, comfort formulae, anti-reflux formulae, lactose-free formulae, preterm formulae, and soy-based formulae.

Median values, first and third quartiles, and minimum and maximum values were calculated for all analyzed formulae. Statistical analyses were performed using Statistica 13.3 (StatSoft, TIBCO Software Inc., Palo Alto, CA, USA). Figures were prepared using Microsoft Excel 365 (Microsoft Corporation, Redmond, WA, USA).

The detection of several Se species prompted an attempt to estimate the possible origin of these compounds, which was explored through statistical analysis. A total of 142 observations were included in the analysis (after excluding missing data), comprising 125 measurements with detectable concentrations and 17 observations in which the Se species was not detected. Consequently, a two-step analytical approach was applied. First, the presence of organic Se species was analyzed as a binary response variable (0—not detected, 1—detected). Firth’s penalized logistic regression was used to assess the influence of selected factors on the occurrence of Se species and to model the detection probability of SeMet and MeSeCys. Second, to investigate the effects of the selected factors on the concentrations of organic Se species, only observations with detectable levels were considered, and a generalized linear model (GLM) with a Gamma distribution and a log-link function was applied. For each polytomous factor, the most populous category was systematically designated as the reference baseline level. This approach ensures optimal numerical stability during the maximum likelihood estimation and maximizes statistical power by minimizing the standard errors of the estimated parameters across all subsequent pairwise contrasts. Several models with different sets of explanatory variables were evaluated. Based on information criteria (AIC and BIC), the deviance-to-degrees-of-freedom ratio, and cross-validation results, the Gamma generalized linear model with a log-link function provided the best fit to the data and was therefore selected for the final analyses. Statistical inference was conducted at the 0.05 significance level. Moreover, to investigate differences among the levels of categorical predictors within the Gamma GLM, post hoc multiple comparisons were performed using estimated marginal means (EMMs). Pairwise contrasts were evaluated as concentration ratios rather than absolute differences. To control the family-wise error rate at alpha = 0.05, *p*-values for all pairwise comparisons were adjusted using Tukey’s honestly significant difference (HSD) method. All analyses were performed in the R environment.

## 3. Results

### 3.1. Selenium Concentrations in Analyzed Formulae

The Se concentrations in the analyzed formulae (µg/100 kcal) are presented in Table 2. Most of the formulae met the recommended minimum and maximum Se levels (78.4% of the tested products). Among the remaining samples, nine formulae were below the detection limit, Se was detected but remained below the minimum value in eighteen formulae, and five formulae exceeded the maximum permissible Se content. The lowest proportion of formulae with values outside the recommended range was observed among infant formulae, where only approximately 11% showed abnormal Se levels. Notably, none of the formulae for special nutritional purposes exceeded the maximum Se limits. The Se concentrations expressed in µg/kg of analyzed formulae are provided in Supplementary Materials (Table S2).

### 3.2. Estimated Daily Intake of Selenium

Table 3 presents the EDI expressed in µg/day, divided into infant formulae, follow-on formulae and special medical purposes formulae. Formulae for special medical purposes were categorized according to their intended use: for infants aged 0–6 months, 7–12 months, from birth to 12 months, and for preterm infants.

There is no specific adequate intake value established for infants under 6 months of age, and therefore, for infant formulae and EDI calculations, the assessment focused on the potential to exceed the UL. The UL value was exceeded in the first month by one formula, which is also above the maximum level permitted by the EU. In subsequent months, the number of formulae exceeding the UL increased: five formulae in the third month and six in the sixth month. This indicates that even when EU standards for nutrient content are met, the upper level may still be exceeded. The lowest Se concentration expressed in µg/100 kcal, corresponding to an EDI above 45 µg (UL value), was 7.8 µg/100 kcal.

The EDI for follow-on formulae was assessed in infants aged 7 and 10 months, assuming that the formula provided 45% and 25% of the total daily energy requirement, respectively. At 7 months, only seven formulae met the daily Se requirement, whereas at 10 months none of the formulae provided adequate intake. This underscores the need to introduce Se-containing complementary foods during the complementary feeding period. None of the formulae exceeded the UL value.

In formulae for special medical purposes, the EDI depended on the formula category. In formulae intended for use from birth to 6 months, the UL value was exceeded at 6 months in two preparations. Similarly to infant formulae, these preparations did not exceed the maximum level recommended by the EU (Table 2). In formulae intended for feeding from 7 to 12 months, only one preparation had an EDI value above the AI. In formulae intended for feeding from 0 to 12 months, the UL values were exceeded at 3 months in two formulae and at 6 months in six formulae. At 7 months, the EDI values for seven formulae were above the AI. Importantly, the preparations that met the AI for 7-month-old infants also exceeded the UL values calculated for 6 months of age. This suggests that formulae intended for use from birth to 12 months may not adequately meet infants’ Se requirements at specific developmental stages. In formulae intended for preterm infants, none of the analyzed samples exceeded the UL values.

### 3.3. Selenium Speciation

The Se concentrations and Se speciation profiles in the different formulae, determined using ICP MS, are presented in Table 4.

According to the manufacturer’s declaration on the label, Se was added in the form of sodium selenite in 72 formulae and sodium selenate in 49 formulae. The Se form was not specified in 26 formulae; only “selenium” was listed. One formula did not list Se in the ingredients, even though it appeared in the nutritional information. Of the 121 samples in which the Se form was specified, 71.1% were consistent with the manufacturer’s declaration, 22.3% showed discrepancies (including 7.4% with concentrations below the detection limit), and 6.6% contained similar proportions of Se species, making it difficult to identify a dominant form.

Two additional organic forms of Se were also identified: selenomethionine (SeMet) and methylselenocysteine (MeSeCys). The mean content of SeMet was 7.3% (min.—0%, max.—27.3%), and that of MeSeCys was 8.5% (min.—0%, max.—29.5%).

As Se species were reported as percentage contributions rather than absolute concentrations, the intake of individual Se forms was estimated using the previously calculated total EDI for 6-month-old infants, the age group with the highest overall Se intake. Using this approach, the estimated mean SeMet intake was 2.3 µg/day, while the estimated mean MeSeCys intake was 1.8 µg/day.

### 3.4. Associations Between Selenium Species and Milk Composition

According to EU and FDA regulations, Se added to formulae for infants must be provided in inorganic forms. Organic Se species may nonetheless appear in the final product because raw materials of biological origin can naturally contain trace amounts of these compounds. To explore the potential origin of the detected Se species, a series of statistical models was applied. A complete overlap in zero observations, where the absence of SeMet and MeSeCys occurred simultaneously in the exact same samples, was noted. Consequently, a shared Firth’s penalized logistic regression was evaluated, revealing that the type of infant formula had no statistically significant effect on the probability of occurrence for either organic Se species (Penalized Likelihood Ratio Test: *χ*^2^ = 7.27, *p* = 0.609), indicating that the occurrence of SeMet and MeSeCys was not associated with the type of formula for infants. The second step, which analyzed only the positive concentration values, evaluated the impact of formula type on the amounts of individual organic Se forms. The statistical analysis showed that the amino acid-based formulae had a significantly lower SeMet level (approximately 76% lower), whereas extensively hydrolyzed formulae exhibited a significantly higher level (approximately 47% higher) than the reference group (infant formulae) (Table 5). No significant differences were observed for the remaining formula categories (Table 5). Furthermore, pairwise comparisons among formula groups (Table 6 and Table 7) revealed that infant formulae and follow-on formulae had markedly higher SeMet concentrations than the amino acid-based formulae, exceeding their levels by more than fourfold and 3.6-fold, respectively. The amino acid-based formula exhibited the lowest SeMet concentration among all groups analyzed. Compared with typical commercial formula categories (anti-reflux, comfort, goat-based, extensively hydrolyzed, lactose-free, and soy-based formulae), SeMet concentrations in the amino acid-based formulae represented only 16% to 28% of the corresponding values observed in these products.

For MeSeCys, the amino acid-based formulae demonstrated a lower level (approximately 60% lower) than infant formulae, while the comfort formulae had approximately 37% lower MeSeCys content compared with the reference group (Table 8). Additionally, pairwise comparisons of estimated marginal means (Table 6) revealed only one statistically significant difference, with the concentration of methylselenocysteine in the amino acid-based formulae corresponding to approximately 27% of that observed in the lactose-free formulae (ratio = 0.266, *p* = 0.0329).

In the case of the analyzed ingredients, no significant effects on the levels of either organic Se form were observed (Table 9 and Table 10), with milk powder, whey, casein, and probiotic selected as the reference baseline levels.

## 4. Discussion

This investigation is based on an extensive set of milk samples and employs a comprehensive Se speciation workflow. The combination of broad sampling and molecular-level characterization provides a novel and methodologically robust contribution to current research on Se distribution in infant formulae intended for infant nutrition. The composition of infant formula is continually modified to more closely match human milk, which underscores the essential need for regular monitoring of nutrient concentrations.

Our study determined the Se concentration in a comprehensive set of powdered milk formulae for infants available on the Polish market intended for consumption from birth to 12 months of age. An additional objective was to identify the chemical forms of Se present in these products. Considering the occurrence of various Se species, including SeMet and MeSeCys, an attempt was made to estimate which raw materials used in formula production could be potential sources of these forms.

The Se concentrations reported in our study were comparable to those described by other authors. Both insufficient Se concentration and formulae for which the calculated EDI may exceed the UL were observed [31,32,33,34,35,36,37]. Some of the available studies focused on comparing different formula types in relation to their Se concentration, whereas others referred to international guidelines (EU, Codex Alimentarius or FDA) specifying the required Se levels in formulae or assessed the estimated daily Se intake. In the study by Pandelova M. et al., 42 formulae for infants from several European countries were analyzed. The mean Se concentrations in infant formulae were 0.09 µg/g, 0.12 µg/g, and 0.07 µg/g for cow-based, soy-based, and hypoallergenic formulae, respectively. For follow-on formulae, the corresponding values were 0.51 µg/g, 0.22 µg/g, and 0.41 µg/g. In some formulae, the estimated daily intake exceeded the UL of 60 µg/day [31]. In the studies of Sola-Larraña et al., which included 105 formulae from Spain, Se concentrations were 7.4 ± 5.6 µg/L in infant formulae, 7.4 ± 5.2 µg/L in follow-up formulae, 9.7 ± 6.3 µg/L in formulae for preterm infants, 7.9 ± 7.9 µg/L in formulae for special medical purposes, and 22.3 ± 12.8 µg/L in soy-based formulae [32]. In Sweden, Ljung C. et al. analyzed nine preparations. They reported Se concentrations of 5.7 and 5.9 µg/L in organic cow-based formulae, 16.0–18.0 µg/L in conventional cow-based formulae, 29.0 µg/L in hydrolysed formula, 18.0 µg/L in casein-extensively hydrolysed formula, 13.0 µg/L in whey-extensively hydrolysed formula, 15.0 µg/L in rice-based formula, and 15.0 µg/L in soy-based formula [33]. In Italy, Bargellini A. et al. analyzed 35 infant formulae mean Se concentration was 13.27 µg/L, with values of 13.82 µg/L in infant formulae, 13.7 µg/L in formulae for allergic infants (milk proteins or lactose-intolerant), and 11.4 µg/L in formulae intended for infants with gastrointestinal problems such as diarrhea or reflux. All values were consistent with EU requirements, and no statistically significant differences in Se concentrations were observed between product categories [34]. In Jordan, Tahboub Y. R. et al. reported a mean Se concentration of 0.12 ± 0.07 µg/g in 22 infant formulae, which was substantially higher than the levels found in human milk, which served as the reference material in that study [35]. In southern China, Lin X. et al. analyzed 54 formulae, in which Se concentrations ranged from 0.07 to 0.32 µg/g, with some values below the Codex Alimentarius reference range [36]. In Poland, Chajduk E. et al. analyzed six formulae for infants. They reported Se concentrations of 0.12–0.20 µg/g in cow-based formulae, 0.16 µg/g in a goat milk-based formula, and a markedly higher value of 0.98 µg/g in a soy-based formula [37]. Differences in Se concentration in studies may result from several factors. The composition of formulae for infants varies with the types of products available on the market and with the choices researchers make when selecting samples. Additional variation may arise from the analytical methods used in different studies.

Our analysis indicates that, regardless of formula type, both Se deficiency and excessive intake are possible. Therefore, regular monitoring of selenium concentrations in infant formulae is important, as inappropriate Se content may carry meaningful health implications. Both insufficient and excessive intakes warrant attention, although the present findings should be interpreted as a screening-level indication that certain products may require closer oversight rather than evidence of clinical toxicity. It should also be noted that the risk of either insufficient or excessive Se intake depends on multiple factors, including the duration of formula consumption, preparation accuracy, type and amount of complementary foods, infant body weight, and Se bioavailability.

Exceeding the UL based on the EDI was already observed in the first month of life, and its frequency increased with infant age up to 6 months. It should be emphasized that this occurred in a small proportion of samples, with approximately 5% exceeding the UL by 6 months of age. Nevertheless, for infants consuming these specific formulae, this represents a potential risk.

In the second half of infancy (beyond 6 months of age), no exceedance of the UL value was observed. This is primarily because infant formulas constitute a much smaller proportion of total energy intake at this stage, as complementary feeding becomes established. Our analysis showed that the EDI for Se provided solely by formulae during this period is often lower than the AI, which highlights the importance of introducing Se-containing complementary foods. Good dietary sources of Se at this age include fish, egg yolk, and broccoli [38].

The next stage of the study was to identify the Se species present in the formulae. The dominant forms were sodium selenite and sodium selenate among the identified Se species, and in most of the analyzed products, their presence was consistent with the manufacturer’s label declaration. However, most products were tested using a single commercial container; the assessment of agreement or disagreement with the declared composition should be interpreted with caution and treated as indicative rather than definitive. Trace amounts of SeMet and MeSeCys were also detected. The estimated intake from these species appears low relative to total Se intake. It is also important to emphasize that reference values for Se speciation have not yet been established. Their occurrence is most likely related to the presence of these species in raw materials such as milk powder, whey, casein, and probiotic cultures. Accordingly, an attempt was made to identify which ingredients used in production could be potential sources of these compounds. The statistical analysis showed that the levels of SeMet and MeSeCys were significantly lower in analog formulae compared with infant formulae. This may be associated with the substantial degree of ingredient fragmentation and the more detailed composition characteristic of these products. Interestingly, SeMet content was significantly higher in extensively hydrolyzed formulae. In the case of MeSeCys, its concentration was also significantly lower in comfort formulae. These differences may therefore be related not to the formula type itself, but rather to the specific added ingredients. However, the analysis of potential components that could contain trace amounts of the examined Se species did not reveal any significant associations. This can likely be explained by the varying proportions of added ingredients and the diversity of ingredient forms. For example, whey powder could be fully demineralized, partially demineralized, or appear in two different forms within the same product, while in other cases only its presence was indicated. These findings highlight the need for further investigation of individual raw materials and a closer examination of production technologies to identify the source of trace amounts of organic Se species.

In addition, evidence from previous studies indicates that several technological and biochemical factors may contribute to the variability observed across products. Variability in raw materials such as milk powder, whey, and casein, together with differences in pH, thermal treatment, enzymatic hydrolysis, and interactions with proteins during processing, can influence Se speciation [39,40,41,42]. Furthermore, the stability of selenite and selenate under industrial conditions may vary, and certain bacterial cultures have been shown to promote the conversion of inorganic Se forms into trace amounts of organic species [43]. These mechanisms may therefore explain why trace organic Se forms can appear even when they are not intentionally added and why their levels do not consistently correlate with specific ingredients.

Due to current regulatory regimes for infant formulae, manufacturers typically monitor total Se rather than its speciation. Establishing regulatory guidance that recommends speciation analysis appears to be beneficial. Such guidance should encourage monitoring not only of finished infant products but also of raw materials, which may exhibit substantial variability, and should facilitate the detection of unintended transformations of Se species during processing. Establishing clearer regulatory expectations could support industrial practice by promoting speciation-focused control of raw materials and processing steps and by defining standardized analytical requirements that enhance consistency between declared and actual Se composition.

A limitation of the study is that it covered only formulae for infants available on the Polish market and did not take into account products from other European countries. In addition, different batches of the same formula were not included for all samples due to the costs associated with purchasing additional units and conducting chemical analyses. As a result, most products were analyzed from a single commercial container, which limits the ability to assess batch-to-batch variability; this constraint also affects the interpretation of non-organic Se species and agreement with manufacturer-declared composition, which should therefore be regarded as indicative rather than definitive. Furthermore, the study estimated Se intake rather than measuring infant Se status, did not assess Se bioavailability, did not incorporate complementary foods into exposure modeling, and could not determine the origin of organic Se species without access to raw materials or production-process samples.

## 5. Conclusions

In most cases, the Se concentration in the analyzed infant formulae was within the recommended range. Nevertheless, the estimated daily intake indicated exceedances of the UL in several samples, highlighting the need for regular monitoring of Se concentrations in formulae for infants, as inappropriate Se content may have significant health implications. The detection of SeMet and MeSeCys provides additional insight into the overall Se profile of these products and opens new avenues for research into Se speciation in formulae for infants. The findings of this study may offer valuable guidance for industry and support the development of regulatory recommendations concerning Se concentration in infant formulae.

## Figures and Tables

**Table 1:** Validation parameters of Se speciation by HPLC-ICP MS and total Se determination by ICP MS.

Parameters	Se IV	Se VI	SeMet	MeSeCys	Total Se in Extract	Total Se
Linearity coefficient R^2^	0.9944	0.9938	0.9994	0.9938	0.9998	0.9997
Analytical range [μg/L]	0.5–50	0.5–50	0.5–50	0.5–50	0.5–50	0.5–50
LOD [μg/kg]	1.5	4.9	2.4	1.8	3.7	28
LOQ [μg/kg]	4.9	16	8.0	6.0	12	93
Precision at 10 μg/L [RSD%, n = 7]	16	15	17	14	-	-
Spike recovery in a representative infant formulae sample (spike level: 2.5 μg/L of each Se form) [%]	80	117	110	120	-	-
Recovery relative to certified values [%]:						
ERM-BD 150 (skimmed milk powder)	-	-	-	-	90	117
BC210a (wheat flour)	-	-	47	-	106	102

**Table 2:** Selenium concentrations in analyzed formulae.

Analyzed Elements	Norms *	Infant Formulae (n = 37)	Follow-On Formulae (n = 33)	Formulae for Special Medical Purposes Intended for Infants (n = 78)	Total Analyzed Formulae (n = 148)
		Median (1st–3rd) <Min–Max>			
Se [µg/100 kcal] *	3–8.6 µg	4.9 (4.0–6.3) <**0.0–15.6**>	4.8 (4.0–6.3) <**0.0–9.3**>	4.0 (3.0–5.5) <**0.0**–8.5>	4.5 (3.3–6.1) <**0.0–15.6**>
Se below minimum levels [amount]		3	5	19	27
Se above maximum levels [amount]		1	4	0	5
Amount of formulae inconsistent with EU regulations [%]		10.8	27.3	24.4	21.6

* Appropriate composition of Se in formulae for infants as specified by the European Union guidelines.

**Table 3:** Estimated daily selenium [µg/day] intake of infants from different formulae.

Analyzed Formulae	Infant Age *				
	1 Month	3 Month	6 Month	7 Month 45% of the Daily Energy Coverage	10 Month 25% of the Daily Energy Coverage
	Median (1st–3rd) <Min–Max>				
Infant formulae (n = 37)	22.3 (14.9–28.2)	28.2 (23.2–36.7)	30.5 (25.2–39.6)		
	<0.0–**71.5**>	<0.0–**90.6**>	<0.0–**97.9**>		
Follow-on formulae (n = 33)				13.6 (11.5–17.9)	8.5 (7.2–11.2)
				<0.0–26.7>	<0.0–16.7>
Formulae for special medical purposes intended for infants (n = 78)					
Intended for infants for birth to 6 months of age (n = 29)	18.2 (13.7–23.3)	23.0 (17.4–29.5)	24.9 (18.8–31.9)		
	<0.0–34.4>	<0.0–43.6>	<0.0–**47.1**>		
Intended for infants from 6 to 12 months of age (n = 21)				11.8 (8.6–14.1)	7.4 (5.4–8.9)
				<0.0–21.5>	<0.0–13.5>
Intended for infants for birth to 12 months of age (n = 24)	21.9 (14.6–32.5)	27.8 (18.6–41.3)	30.0 (20.1–44.6)	13.6 (9.1–20.3)	8.5 (5.7–12.7)
	<0.0–39.1>	<0.0–**49.6**>	<0.0–**53.6**>	<0.0–24.4>	<0.0–15.3>
Intended for preterm infants (n = 4)	15.8 (4.5–24.0)	20.1 (5.8–30.4)	21.7 (6.2–32.9)		
	<0.0–25.4>	<0.0–32.1>	<0.0–34.7>		

* Calculations are provided only for the age ranges for which each formula category is intended according to manufacturer recommendations. Adequate intake for infants 6–11 months—20 µg; upper level for Se for infants 4–6 months—45 µg, for 7–11 months—55 µg.

**Table 4:** Selenium concentrations and selenium speciation in different formulae determined using ICP MS.

Analyzed Formulae	Se [µg/kg] *	Se IV [%]	Se VI [%]	SeMet [%]	MeSeCys [%]	Others [%]
	Median (1st–3rd) <Min–Max>					
Infant formulae (n = 37)	200.5 (160.0–245.5)	10.2 (2.2–55.4)	11.4 (5.8–48.0)	8.7 (5.1–11.5)	7.9 (5.8–10.8)	23.1 (6.9–37.2)
	<0.0–23.8>	<0.0–77.0>	<0.0–76.5>	<0.0–23.8>	<0.0–29.5>	<0.0–94.0>
Follow-on formulae (n = 33)	193.8 (141.0–250.4)	7.7 (4.1–60.6)	12.3 (7.0–55.6)	6.6 (4.6–9.1)	7.1 (4.0–8.7)	19.4 (4.0–47.8)
	<0.0–451.4>	<0.0–89.0>	<0.0–84.1>	<0.0–27.3>	<0.0–26.0>	<0.0–92.6>
Total formulae for special medical intend (n = 78)	163.9 (121.0–234.2)	20.6 (4.8–54.0)	5.0 (0.0–14.5)	9.4 (3.0–13.2)	6.3 (3.0–9.8)	21.8 (6.3–56.3)
	<0.0–362.0>	<0.0–94.1>	<0.0–87.9>	<0.0–24.8>	<0.0–26.2>	<0.0–100.0>
Extensively hydrolyzed formulae (n = 26)	163.9 (129.5–211.1)	42.6 (8.4–62.3)	3.3 (0.0–8.0)	12.2 (7.7–15.2)	6.2 (4.4–9.5)	15.1 (1.3–42.1)
	<0.0–336.8>	<0.0–82.4>	<0.0–71.1>	<0.0–24.8>	<0.0–14.4>	<0.0–91.6>
Amino acid-based formulae (n = 6)	199.9 (172.3–340.0)	44.4 (22.1–63.7)	2.8 (0.0–9.4)	1.4 (0.0–3.0)	0.9 (0.0–4.8)	41.4 (21.9–77.9)
	<156.1–362.0>	<19.1–94.1>	<0.0–11.2>	<0.0–3.5>	<0.0–9.0>	<0.0–80.9>
Formulae manufactured from goats’ milk proteins (n = 15)	159.4 (76.5–236.6)	14.6 (6.2–34.5)	5.9 (3.3–14.7)	10.5 (7.8–12.4)	7.3 (5.9–8.1)	44.0 (22.6–58.2)
	<0.0–310.0>	<0.0–78.7>	<0.0–58.6>	<0.0–14.5>	<0.0–16.5>	<0.0–71.0>
Comfort-formulae (n = 12)	182.4 (91.7–272.5)	2.2 (0.0–27.4)	5.2 (0.0–73.2)	8.2 (0.0–11.6)	3.2 (0.0–5.2)	8.6 (2.3–26.8)
	<0.0–301.9>	<0.0–73.8>	<0.0–87.9>	<0.0–13.3>	<0.0–13.4>	<0.0–100.0>
Anti-reflux formulae (n = 7)	135.0 (101.5–187.5)	45.2 (8.6–56.8)	8.2 (0.0–11.8)	10.3 (5.0–18.1)	10.3 (5.6–17.0)	16.4 (0.0–79.0)
	<96.8–213.3>	<0.0–76.3>	<0.0–12.9>	<0.0–24.3>	<0.0–17.7>	<0.0–91.6>
Lactose-free formulae (n = 5)	153.4 (147.9–183.4)	34.5 (4.9–52.1)	27.6 (15.9–43.2)	8.9 (6.4–9.4)	10.9 (9.8–14.6)	15.7 (9.5–22.4)
	<117.4–274.8>	<2.6–54.1>	<4.3–58.2>	<4.5–13.8>	<8.1–26.2>	<8.5–33.9>
Preterm formulae (n = 4)	167.0 (53.4–271.0)	27.5 (6.0–53.7)	1.1 (0.0–15.8)	3.8 (0.8–9.3)	6.8 (0.9–13.1)	50.2 (8.0–92.2)
	<0.0–314.7>	<0.0–64.2>	<0.0–29.4>	<0.0–12.7>	<0.0–14.7>	<0.1–100.0>
Soya-based formulae for infants (n = 3)	257.1 (0.0–296.7)	9.1 (0.0–52.5)	0.0 (0.0–4.8)	2.9 (0.0–16.3)	1.5 (0.0–8.5)	17.9 (0.0–86.5)
	<0.0–296.7>	<0.0–52.5>	<0.0–4.8>	<0.0–16.3>	<0.0–8.5>	<0.0–86.5>
Total analised formulae (n = 148)	187.2 (125.2–239.7)	15.5 (3.4–54.5)	8.0 (2.4–30.7)	8.5 (4.4–12.2)	6.9 (3.9–10.0)	21.1 (5.0–47.1)
	<0.0–451.4>	<0.0–94.1>	<0.0–87.9>	<0.0–27.3>	<0.0–29.5>	<0.0–100.0>

SeMet—Selenomethionine; MeSeCys—Methylselenocysteine. * total Se concentrations in extract.

**Table 5:** Effect of formula type on SeMet content: GLM results (Gamma distribution, log link).

Analyzed Parameter	Coefficients:			
	Estimate	Std. Error	t Value	*p*
(Intercept)	2.269848	0.088399	25.677	0.0000 ***
Follow-on formulae	−0.147286	0.127082	−1.159	0.2489
Amino acid-based formulae	−1.429801	0.265198	−5.391	0.0000 ***
Anti-reflux formulae	0.303400	0.222467	1.364	0.1753
Comfort-formulae	0.049937	0.197667	0.253	0.8010
Formulae manufactured from goats’ milk proteins	0.073105	0.160237	0.456	0.6491
Extensively hydrolyzed formulae	0.382974	0.142539	2.687	0.0083 **
Lactose-free formulae	−0.141682	0.265198	−0.534	0.5942
Preterm formulae	−0.354952	0.301941	−1.176	0.2422
Soya-based formula for infants	−0.008633	0.364479	−0.024	0.9811

*** *p* < 0.001, ** *p* < 0.01; (Dispersion parameter for Gamma family taken to be 0.2500616).

**Table 6:** Estimated marginal means of conditional Selenomethionine (SeMe) and Methylselenocysteine (MeSeCys) concentrations across the investigated infant formula groups.

Formula Group	Estimated Mean		Std. Error		Lower 95% CI		Upper 95% CI	
	SeMe	MeSeCys	SeMe	MeSeCys	SeMe	MeSeCys	SeMe	MeSeCys
Infant formulae	9.68	9.88	0.856	0.968	8.12	8.14	11.5	12.00
Follow-on formulae	8.35	8.07	0.763	0.817	6.97	6.61	10.0	9.87
Amino acid-based formulae	2.32	3.91	0.579	1.080	1.41	2.26	3.8	6.77
Anti-reflux formulae	13.11	11.96	2.680	2.710	8.75	7.64	19.6	18.73
Comfort formulae	10.17	6.24	1.800	1.220	7.17	4.23	14.4	9.204
Goat-based formulae	10.41	8.29	1.390	1.230	7.99	6.18	13.6	11.11
Extensively hydrolyzed formulae	14.19	8.01	1.590	0.992	11.37	6.26	17.7	10.24
Lactose-free formulae	8.40	14.67	2.100	4.060	5.12	8.47	13.8	25.40
Preterm formulae	6.79	9.40	1.960	3.010	3.83	4.99	12.0	17.72
Soy-based formulae	9.59	5.03	3.390	1.970	4.76	2.32	19.3	10.94

Confidence level = 95%. Confidence intervals were back-transformed from the log scale.

**Table 7:** Post hoc pairwise comparisons (Tukey’s HSD) showing only statistically significant differences (*p* < 0.05) in SeMe across infant formula groups.

Contrast	Ratio	Std. Error	*p*
Infant formulae/amino acid-based formulae	4.18	1.11	0.0000 ***
Follow-on formulae/amino acid-based formulae	3.61	0.96	0.0002 ***
Follow-on formulae/extensively hydrolyzed formulae	0.59	0.08	0.0130 *
Amino acid-based formulae/anti-reflux formulae	0.18	0.06	0.0000 ***
Amino acid-based formulae/comfort formulae	0.23	0.07	0.0002 ***
Amino acid-based formulae/goat-based formulae	0.22	0.06	0.0000 ***
Amino acid-based formulae/extensively hydrolyzed formulae	0.16	0.04	0.0000 ***
Amino acid-based formulae/lactose-free formulae	0.28	0.10	0.0143 *
Amino acid-based formulae/soy-based formulae	0.24	0.10	0.0429 *

*** *p* < 0.001, * *p*< 0.05.

**Table 8:** Effect of formula type on MeSeCys content: GLM results (Gamma distribution, log link).

Analyzed Parameter	Coefficients:			
	Estimate	Std. Error	t Value	*p*
(Intercept)	2.29049	0.09799	23.375	0.0000 ***
Follow-on formulae	−0.20189	0.14087	−1.433	0.1546
Amino acid-based formulae	−0.92733	0.29397	−3.154	0.0021 **
Anti-reflux formulae	0.19116	0.24660	0.775	0.4399
Comfort-formulae	−0.45952	0.21911	−2.097	0.0382 *
Formulae manufactured from goats’ milk proteins	−0.17580	0.17762	−0.990	0.3244
Extensively hydrolyzed formulae	−0.21015	0.15801	−1.330	0.1862
Lactose-free formulae	0.39506	0.29397	1.344	0.1816
Preterm formulae	−0.04991	0.33470	−0.149	0.8817
Soya-based formula for infants	−0.67434	0.40403	−1.669	0.0979

*** *p* < 0.001, ** *p* < 0.01, * *p* < 0.05; (Dispersion parameter for Gamma family taken to be 0.3072698).

**Table 9:** Effect of selected added ingredients on SeMet content: GLM results (Gamma distribution, log link).

Analyzed Parameter	Coefficients:			
	Estimate	Std. Error	t Value	*p*
(Intercept)	2.1921101	0.0834801	26.259	0.000 ***
Addition of whey	−0.0241901	0.1305684	−0.185	0.853
No addition of whey	−0.0006517	0.1264469	−0.005	0.996
Addition of probiotics	0.1708465	0.1048298	1.630	0.106
No addition of milk powder	0.1497368	0.1216127	1.231	0.221
Addition of casein	0.3070848	0.2781958	1.104	0.272

*** *p* < 0.001; (dispersion parameter for Gamma family taken to be 0.2693011).

**Table 10:** Effect of selected added ingredients on MeSeCys content: GLM results (Gamma distribution, log link).

Analyzed Parameter	Coefficients:			
	Estimate	Std. Error	t Value	*p*
(Intercept)	2.302540	0.090238	25.516	0.000 ***
Addition of whey	−0.222779	0.141139	−1.578	0.117
No addition of whey	−0.175681	0.136684	−1.285	0.201
Addition of probiotics	−0.001644	0.113316	−0.015	0.988
No addition of milk powder	−0.066273	0.131458	−0.504	0.615
Addition of casein	−0.091015	0.300718	−0.303	0.763

*** *p* < 0.001; (dispersion parameter for Gamma family taken to be 0.3146695).

## Data Availability

The original contributions presented in this study are included in the article/Supplementary Materials. Further inquiries can be directed to the corresponding author.

## Supplementary Materials

The following supporting information can be downloaded at: https://www.mdpi.com/article/10.3390/foods15173007/s1, Table S1: Instrumental parameters for the determination of selenium species and total elements; Table S2: Selenium concentrations in analyzed formulae [µg/g]; Table S3. Basic characteristics of the analyzed samples.
