# Supplementary material for: Selenium Concentration and Speciation in Formulae for Infants: Implications for Infant Health
Source: Foods. 2026 Aug 26;15(17):3007. doi: 10.3390/foods15173007 (PMC13564777; doi:10.3390/foods15173007)
Supplement: Supplementary file 1 [file foods-15-03007-s001.zip › foods-4470780-supplementary.pdf]

Table S1. Instrumental parameters for the determination of selenium species and total elements.

**HPLC conditions**				
Pump	AnalytikJena PQ LC			
Column	Hamilton PRP-X100 (4.1 x 50 mm, 5 μm)			
Mobile phase	A: 5 mmol L^{-1} acetate buffer, pH= 4.7			
	B: 150 mmol L^{-1} acetate buffer, pH= 4.7			
Gradient program	Time (min)	A (%)	B (%)	Mobile phase flow rate ($mL\ min^{-1}$)
	Initial	100		1.0
	0.5	50	50	1.0
	1.0		100	1.0
	1.75		100	1.5
	6.5		100	1.5
	7.0	50	50	1.5
	7.5	100		1.5
	9.5	100		1.0
Injection volume (μl)	100			
Column temperature (°C)	20			

**ICP MS conditions**	**for total Se**	**for speciation analysis**
Spectrometer	AnalytikJena Plasma Quant MS Q	
Radio frequency power (kW)	1.20	1.20
Nebulizer flow rate ($L\ min^{-1}$)	0.97	0.97
Plasma flow rate ($L\ min^{-1}$)	9.0	9.0
Auxiliary flow rate ($L\ min^{-1}$)	1.5	1.5
Sampling depth (mm)	5.0	5.0
Spray chamber temperature (°C)	3	3
Dwell time (μs)	20000	50000
iCRC mode	H_2 (80 $mL\ min^{-1}$)	H_2 (120 $mL\ min^{-1}$)
Skimmer bias (V)	-	2
Nebulizer pump (rps)	12	35
Monitored isotope (m/z)	^{78}Se, ^{82}Se,	^{78}Se, ^{82}Se,
Internal standard	Sc, Y, Rh (5.0 μg L^{-1})	Ir, Rh (2.5 μg L^{-1})

Table S2. Selenium concentrations in analyzed formulae [μg/g].

**Analyzed element**	**Infant formulae (n=37)**	**Follow-on formulae (n=33)**	**Formulae for special medical purposes intended for infants (n=78)**	**Total analyzed formulae (n=148)**
	Median (1st–3rd)<br><min–max>			
Se [μg/g]	0.25 (0.20-0.31)<br><0.00-0.80>	0.23 (0.19-0.30)<br><0.00-0.46>	0.20 (0.15-0.28)<br><0.00-0.40>	0.22 (0.16-0.30)<br><0.00-0.80>

Table S3. Basic characteristics of the analyzed samples.

Sample number	Type of analyzed formulae	Subtype of analyzed formulae	Se [μg/kg]
1	infant formulae	-	240
2	follow-on formulae	-	220
3	infant formulae	-	160
4	special formulae	comfort formulae	190
5	special formulae	comfort formulae	<DL
6	special formulae	anti-reflux formulae	150
7	special formulae	extensively hydrolyzed formulae	98
8	special formulae	extensively hydrolyzed formulae	350
9	infant formulae	-	280
10	follow-on formulae	-	240
11	special formulae	preterm formulae	<DL
12	special formulae	extensively hydrolyzed formulae	160
13	special formulae	extensively hydrolyzed formulae	200
14	special formulae	extensively hydrolyzed formulae	190
15	special formulae	extensively hydrolyzed formulae	340
16	special formulae	extensively hydrolyzed formulae	200
17	special formulae	extensively hydrolyzed formulae	110
18	follow-on formulae	-	190
19	follow-on formulae	-	<DL
20	infant formulae	-	300
21	infant formulae	-	100
22	infant formulae	-	260
23	follow-on formulae	-	100
24	special formulae	goat-based formulae	140
25	special formulae	goat-based formulae	150
26	special formulae	goat-based formulae	140
27	follow-on formulae	-	190
28	special formulae	anti-reflux formulae	170
29	special formulae	comfort formulae	110
30	special formulae	lactose-free formulae	200
31	special formulae	anti-reflux formulae	200
32	infant formulae	-	180
33	infant formulae	-	800
34	special formulae	goat-based formulae	250
35	follow-on formulae	-	140
36	special formulae	goat-based formulae	150
37	infant formulae	-	170
38	special formulae	lactose-free formulae	250
39	infant formulae	-	290
40	special formulae	extensively hydrolyzed formulae	<DL
41	follow-on formulae	-	430
42	special formulae	extensively hydrolyzed formulae	210
43	special formulae	comfort formulae	120
44	special formulae	anti-reflux formulae	160
45	special formulae	comfort formulae	<DL

46	special formulae	soya-based formulae	<DL
47	special formulae	comfort formulae	100
48	infant formulae	-	270
49	follow-on formulae	-	280
50	special formulae	lactose-free formulae	170
51	infant formulae	-	420
52	follow-on formulae	-	110
53	special formulae	extensively hydrolyzed formulae	160
54	special formulae	extensively hydrolyzed formulae	270
55	infant formulae	-	400
56	follow-on formulae	-	470
57	special formulae	amino acid-based formulae	300
58	special formulae	extensively hydrolyzed formulae	160
59	special formulae	extensively hydrolyzed formulae	150
60	special formulae	amino acid-based formulae	200
61	special formulae	preterm formulae	100
62	special formulae	goat-based formulae	150
63	special formulae	lactose-free formulae	130
64	infant formulae	-	310
65	follow-on formulae	-	290
66	special formulae	comfort formulae	280
67	infant formulae	-	200
68	follow-on formulae	-	450
69	infant formulae	-	210
70	special formulae	extensively hydrolyzed formulae	170
71	follow-on formulae	-	300
72	infant formulae	-	140
73	follow-on formulae	-	230
74	infant formulae	-	220
75	infant formulae	-	330
76	special formulae	preterm formulae	270
77	special formulae	comfort formulae	310
78	special formulae	anti-reflux formulae	170
79	special formulae	extensively hydrolyzed formulae	<DL
80	special formulae	extensively hydrolyzed formulae	190
81	special formulae	extensively hydrolyzed formulae	170
82	infant formulae	-	420
83	follow-on formulae	-	200
84	infant formulae	-	<DL
85	follow-on formulae	-	120
86	special formulae	extensively hydrolyzed formulae	280
87	special formulae	amino acid-based formulae	160
88	special formulae	amino acid-based formulae	320
89	follow-on formulae	-	170
90	infant formulae	-	230
91	special formulae	goat-based formulae	<DL
92	special formulae	goat-based formulae	95
93	infant formulae	-	200

94	infant formulae	-	170
95	follow-on formulae	-	150
96	infant formulae	-	250
97	follow-on formulae	-	270
98	special formulae	extensively hydrolyzed formulae	160
99	special formulae	comfort formulae	370
100	follow-on formulae	-	430
101	special formulae	amino acid-based formulae	400
102	infant formulae	-	230
103	infant formulae	-	290
104	special formulae	anti-reflux formulae	120
105	special formulae	goat-based formulae	250
106	special formulae	goat-based formulae	320
107	infant formulae	-	310
108	infant formulae	-	310
109	follow-on formulae	-	390
110	special formulae	goat-based formulae	280
111	infant formulae	-	200
112	follow-on formulae	-	230
113	follow-on formulae	-	210
114	infant formulae	-	210
115	infant formulae	-	300
116	special formulae	anti-reflux formulae	230
117	special formulae	extensively hydrolyzed formulae	270
118	follow-on formulae	-	380
119	special formulae	extensively hydrolyzed formulae	240
120	follow-on formulae	-	160
121	infant formulae	-	420
122	special formulae	extensively hydrolyzed formulae	350
123	special formulae	extensively hydrolyzed formulae	230
124	follow-on formulae	-	390
125	special formulae	goat-based formulae	250
126	special formulae	comfort formulae	270
127	infant formulae	-	340
128	special formulae	goat-based formulae	230
129	follow-on formulae	-	210
130	follow-on formulae	-	210
131	follow-on formulae	-	210
132	infant formulae	-	160
133	follow-on formulae	-	240
134	special formulae	soya-based formulae	370
135	special formulae	comfort formulae	340
136	special formulae	lactose-free formulae	380
137	special formulae	amino acid-based formulae	230
138	special formulae	goat-based formulae	220
139	special formulae	soya-based formulae	350
140	follow-on formulae	-	340
141	special formulae	comfort formulae	360

142	special formulae	extensively hydrolyzed formulae	360
143	special formulae	extensively hydrolyzed formulae	380
144	special formulae	preterm formulae	240
145	special formulae	goat-based formulae	330
146	infant formulae	-	340
147	follow-on formulae	-	270
148	infant formulae	-	210
